# Effectiveness of Certolizumab Pegol in Treating Rheumatoid Arthritis Patients with Persistent Inflamed Residual Mono- or Oligosynovitis Resistant to Prior TNF-*α* Inhibitors

**DOI:** 10.1155/2015/348614

**Published:** 2015-09-14

**Authors:** Syuichi Koarada, Masahiko Tsuboi, Mitsunori Komine, Yoshinobu Nakao, Yukiko Tokuda, Yukihide Ono, Satoko Tashiro, Akihito Maruyama, Nobuyuki Ono, Akihide Ohta, Yoshifumi Tada

**Affiliations:** ^1^Division of Rheumatology, Faculty of Medicine, Saga University, 5-1-1 Nabeshima, Saga 849-8501, Japan; ^2^Nagasaki Medical Hospital of Rheumatology, 1-21 Aburaya-machi, Nagasaki 850-0832, Japan; ^3^Tsuruta Rehabilitation Clinic, 174-8 Ogi-Ushizu, Saga 849-0305, Japan

## Abstract

We report four cases of successful treatment with certolizumab pegol (CZP) of rheumatoid arthritis (RA) patients with persistent inflamed residual mono- or oligosynovitis resistant to prior TNF-*α* inhibitors. Although the patients were in a moderate disease activity, a low activity, or a remission of RA, they sustained inflammatory mono-/oligoarthritis even after treatment with prior TNF inhibitors. They were then all treated with CZP and observed in a serial ultrasonography. In all cases, the positive power Doppler signals in the joint have disappeared promptly and all of the patients were able to retain remission in the long term. The treatment of CZP to the refractory mono-/oligoarthritis of inflammatory synovitis in RA patients has not been previously described. The cases suggest that it may be associated with the feature of CZP, possible effective penetration into the site of inflammation.

## 1. Introduction

In rheumatoid arthritis (RA), persistent inflamed mono- or oligoarthritis is a frequent clinical problem and is difficult to treat [1, 2]. Even in clinical remission (CR), the prevalence of ultrasound- (US-) detected residual synovitis in patients with RA is frequent [3]. Importantly, residual synovitis is the risk of relapse and structural progression in RA patients with CR by the disease-modifying antirheumatic drugs (DMARDs), including biological therapies [1, 3]. We report four cases of successful treatment with certolizumab pegol (CZP) of RA patients with persistent inflamed residual mono- or oligosynovitis resistant to prior TNF-*α* inhibitors.

## 2. Case Presentation

A representative case was a 27-year-old Japanese woman with four-year history of active RA who complained in February 2013 of pain and swelling of the bilateral wrists, shoulders, and foots (case 1). She had been already treated with methotrexate (MTX) 10 mg/week orally. Because she was referred to our clinic with severe polyarthritis, a combination of golimumab administered subcutaneously (SC) and methotrexate (MTX) 10 mg/week orally was medicated. Her symptoms other than the left wrist have quickly disappeared. On clinical examination after a year, although systemic polyarthritis was remarkably improved and she obtained CR in DAS28-CRP (Disease Activity Score 28-CRP), evident monosynovitis of the left wrist had persisted. She complained of restriction of wrist movements for a month. She was referred to a near orthopedic doctor for synovectomy. However, she did not agree on surgical therapy. US imaging showed the persistent left wrist synovitis (PD-positive; grade 2) (baseline). TNF inhibitor was switched to CZP. Her symptom of the left wrist was resolved after 2 weeks and her PD signals in US have disappeared completely after 2 months (PD-negative; grade 0). Since the improvement of clinical symptoms and laboratory data, the administration of MTX was tapered off and CZP was stopped after 8 months. Presently, she achieved drug-free remission for 5 months.

**Figure 1:**
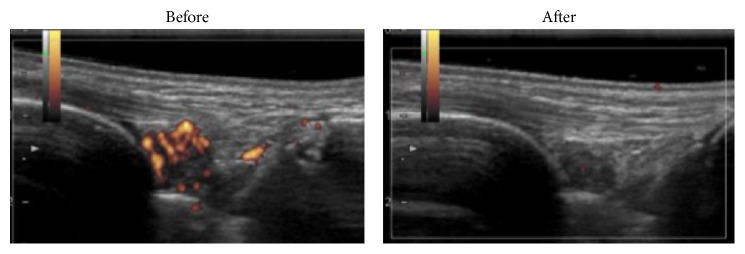
A representative result of US of the wrist of case 3 demonstrated PD-positive synovitis before treatment with CZP. US showed disappearance of PD-positive inflammatory synovitis after treatment with CZP.

We experienced other three patients with RA who had sustained inflammatory mono- or oligoarthritis even after treatment with prior TNF inhibitors. The patients were all female with a mean (SD) age of 42.3 (14.5) years and a mean (SD) disease duration of 6.0 (4.8) years. A mean (SD) period of treatment with prior TNF inhibitors was 24.8 (16.8) months. In US, all patients had power Doppler- (PD-) positive synovitis. They were then all treated with CZP and observed in a serial US. Each patient had a physical and laboratory evaluation before and after treatment. All patients responded well after the injection of CZP as evaluated by the reduction in the number of swollen and tender joints (Table 1). In all cases, the PD-positive signals in the joints were not detected after treatment (a mean (SD) duration of 1.9 (0.9) months). The effectiveness was associated with the improvement of US findings. The DAS28-CRP score (mean (SD); 2.69  (0.68) → 1.55  (0.34)) and PD grade (2 → 0) decreased (Figure 1 and Table 1). The effectiveness was associated with the improvement of US findings. No adverse reactions were noted. All of the patients were able to retain remission in the long term with drug off (case 1; 13 months) or with remaining CZP (case 2; 14 months, case 3; 7 months, and case 4; 4 months).

## 3. Discussion

Persistent mono- or oligoarthritis may be even destructive and necessitate joint replacement surgery in the future. Therefore, surgical synovectomy and intra-articular (IA) steroids, chemical, and tumor necrosis factor-*α*- (TNF-*α*-) inhibitors are local therapeutic options in RA resistant to other medical treatments [1, 4]. However, the efficacy of local therapy is not always evident and it may be of short duration [1]. Moreover, injection site reactions [5] and septic arthritis secondary to IA TNF inhibitors [6] have been experienced. IA therapy of TNF inhibitors has not become a mainstay in RA [7]. In this situation, a pharmacological effect of agent at the site of inflammation may be an important factor for the effective treatment of residual arthritis. Most soluble agents are rapidly absorbed from the inflamed joint to systemic circulation and the transience is a major obstacle to attain clinical effect. Modification of drugs is a strategy to prolong the residence time in the joint [7]. PEGylation (conjugation of a PEG (polyethylene glycol) moiety) is a conventional chemical method to increase the bioavailability of drugs and to delay systemic elimination [8]. This approach has also the potential to increase in the drug residence time in inflamed joints. As shown in an animal model, CZP, PEGylated TNF inhibitor, penetrates inflamed joints more effectively than other TNF inhibitors [9]. However, the evidence of these features of CZP in human has not been clinically confirmed.

CZP is efficacious and well tolerated in phase III clinical trials in RA patients with active disease, irrespective of concomitant or previous therapy [10]. Schiff et al. reported that CZP in RA patients who are secondary nonresponders to TNF inhibitors, demonstrating a good response and a safety profile [11]. However, these patients had active disease defined as ≥6 tender and swollen joints of 28-joint count (polyarthritis). There are no published data relating to the treatment with CZP of refractory mono- and oligoarthritis of inflammatory synovitis resistant to prior TNF inhibitors in RA. Our cases suggested the efficacy of CZP in the patients with RA having residual mono- or oligoarthritis. In conclusion, the clinical benefits may be associated with the drug's feature of localized distribution of CZP compared to other TNF inhibitors. Because potentially effective, safe, and long-lasting treatment for persistent and residual mono- or oligoarthritis is greatly required in clinical practice of RA, further examination in a prospective, randomized, controlled, and multicenter comparative effectiveness study of CZP versus other TNF inhibitors is important to draw rational conclusion.

## Figures and Tables

**Table 1:** Clinical and laboratory changes in patients with RA and mono- or oligoarthritis treated with CZP.

Case	Age/sex	Stage	DD (year)	DMARD	Prior biologics (duration)	NSJ/NTJ prior→before→after	DAS28-CRP prior→before→after	Imaged joint	PD (grade)		
									Before	After	Duration
1	27 F	I	4	MTX	GLM (12 m)	4/3→1/1→0/0	3.87→2.21→1.45	Wrist	2	0	2 m
2	41 F	III	2	BUC	GLM (10 m)	1/3→1/1→0/0	2.34→2.02→1.11	1st IP	2	0	3 m
3	39 F	III	13	MTX	IFX (15 m), ETN (30 m)	2/4→2/2→0/1	3.82→3.46→1.76	Wrist	2	0	1.5 m
4	62 F	II	4	MTX	ETN (32 m)	5/6→3/1→1/0	4.24→3.05→1.88	Elbow	2	0	1 m

Mean	42.3				24.8 m	3/4→1.75/1.25→0.25/0.25	3.56→2.69→1.55		2	0	1.9 m
(SD)	(14.5)				(16.8 m)		(0.84) (0.68) (0.34)				(0.9 m)

DD: disease duration, NSJ/NTJ: the number of swollen and tender joints, m: month(s), F: female, stage: Steinbrocker classification, MTX: methotrexate, BUC: bucillamine, GLM: golimumab, IFX: infliximab, ETN: etanercept. Prior: DAS28 scores prior to treatment with the 1st TNF-inhibitor.

## Data Sharing Statement

Additional data and information about the study can be accessed by contacting the corresponding author.
